# Recurrent Thrombosis in a Very Elderly Patient With Dementia, Atrial Fibrillation, and Idiopathic Thrombocytopenic Purpura on Eltrombopag Treatment

**DOI:** 10.7759/cureus.70792

**Published:** 2024-10-03

**Authors:** Satoshi Kurisu, Hitoshi Fujiwara, Takeshi Shimomura

**Affiliations:** 1 Department of Cardiology, National Hospital Organization Hiroshima-Nishi Medical Center, Otake, JPN; 2 Department of Haematology, National Hospital Organization Hiroshima-Nishi Medical Center, Otake, JPN

**Keywords:** anticoagulation, bleeding, coronary embolism, myocardial infarction, thrombopoietin receptor agonist

## Abstract

Idiopathic thrombocytopenic purpura (ITP) is an autoimmune disorder, leading to an increased bruising and bleeding tendency. Eltrombopag, a thrombopoietin receptor agonist, is an effective treatment option even in elderly patients with ITP. In this report, we present a case of recurrent thrombosis, presenting as acute myocardial infarction and cerebral infarction, in a very elderly patient with dementia, atrial fibrillation, and ITP on eltrombopag treatment. Multiple comorbidities with thrombosis and bleeding risks posed a therapeutic dilemma. Conditions unique to the elderly, such as cognitive impairment or frailty, led to a delayed diagnosis. This case gives an insight into how to manage a practical therapeutic problem in such a case. Physicians need to understand conditions unique to the elderly, such as cognitive impairment, frailty, or quality of life that influence treatment goals. These efforts can help provide health benefits while minimizing treatment risks, especially in elderly patients with multiple comorbidities.

## Introduction

Idiopathic thrombocytopenic purpura (ITP) is an autoimmune disorder characterized by antibody- and cell-mediated destruction of platelets and suppression of platelet production, leading to an increased bruising and bleeding tendency [1-3]. Persistently low platelet counts are associated with an increased risk of serious bleeding such as intracranial hemorrhage or gastrointestinal bleeding [4,5]. The traditional goal of management is to provide a hemostatic platelet count while minimizing treatment-related toxicity. Currently available treatments for ITP include glucocorticoids, splenectomy, immunosuppressants, and thrombopoietin receptor agonists (TPO-RAs). Eltrombopag, a TPO-RA, is an effective treatment option even in elderly patients with ITP, but patients should be closely monitored for response and side effects during treatment [6].

Platelet activation and aggregation as well as fibrin generation are essential in blood coagulation. In clinical practice, anticoagulant drugs are mostly used in atrial fibrillation (AF), whereas antiplatelet drugs are mainstays in the management of acute myocardial infarction (AMI). The use of these drugs leads to an increased risk of bleeding, thereby being a double-edged sword in patients with ITP. It requires careful management with multidisciplinary expertise.

Here, we report a case of recurrent thrombosis, presenting as AMI and cerebral infarction, in a very elderly patient with dementia, AF, and ITP on eltrombopag treatment.

## Case presentation

**Figure 1:**
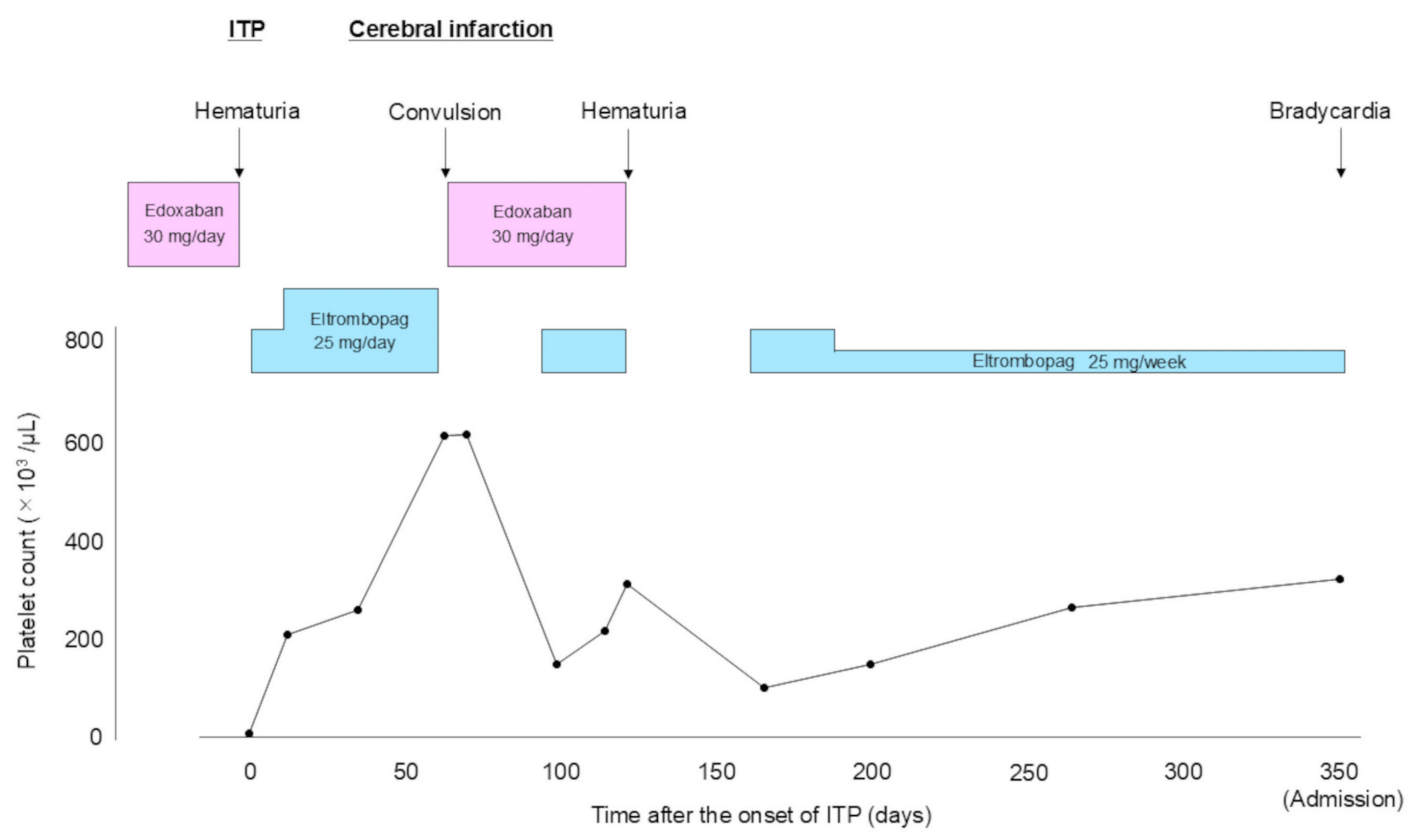
Time course of platelet count after the onset of idiopathic thrombocytopenic purpura. The patient was diagnosed with ITP based on the excessively decreased platelet count. Eltrombopag was introduced for ITP. Two months later, the patient was admitted due to the occurrence of cerebral infarction. Laboratory data indicated a rapid increase in platelet count over four weeks and eltrombopag was discontinued. After the titration of eltrombopag, the patient was transferred to a hospital for rehabilitation with a dose of 25 mg per week. ITP: idiopathic thrombocytopenic purpura

A 93-year-old man with dementia and AF on edoxaban treatment (30 mg/day) presented with hematuria. The patient was diagnosed with ITP based on the excessively decreased platelet count (8 × 10^3^/µL) (reference range: 158-348 × 10^3^/µL). Edoxaban was discontinued and eltrombopag was introduced for ITP (Figure 1). He was followed up on an outpatient basis every one to four weeks. Two months later, the patient was admitted due to the occurrence of cerebral infarction. Laboratory data indicated a rapid increase in platelet count over four weeks and eltrombopag was discontinued. Edoxaban was reintroduced but discontinued again due to hematuria. After the titration of eltrombopag, the patient was transferred to a hospital for rehabilitation with a dose of 25 mg per week. Twelve months later, the patient was found to have bradycardia by nurses, although he had no complaints. He was transferred to our hospital three hours after its recognition. Anticoagulation for AF remained discontinued due to recurrent hematuria.

At the initial presentation, the patient’s pulse rate was 32 beats/minute, blood pressure was 92/60 mmHg, oxygen saturation was 96%, Glasgow coma scale score was E3V3M4, and CHADS2 score was 3 points. No obvious jugular vein distensions were noted. Platelet count (276 × 10^3^/µL) was normal and myocardial enzymes were increased (Table 1).

**Table 1:** Laboratory data. At the initial presentation, platelet count was normal and myocardial enzymes were increased.

Variable	Initial presentation	7 days later	Reference range
White blood cell counts	8,200/µL	6,000/µL	3,300–8,600/µL
Red blood cell counts	4.17 × 10^6^/µL	4.22 × 10^6^/µL	4.35–5.55 × 10^6^/µL
Hemoglobin	12.9 g/dL	13.2 g/dL	13.7–16.8 g/dL
Platelet counts	276 × 10^3^/µL	288 × 10^3^/µL	158–348 × 10^3^/µL
Aspartate aminotransferase	99 U/L	23 U/L	13–30 U/L
Alanine aminotransferase	26 U/L	18 U/L	10–42 U/L
Lactate dehydrogenase	468 U/L	276 U/L	124–222 U/L
Creatine kinase	450 U/L	77 U/L	59–248 U/L
Total protein	7.4 g/dL	-	6.6–8.1 g/dL
Albumin	3.5 g/dL	-	4.1–5.1 g/dL
Blood urea nitrogen	37.6 mg/dL	15.0 mg/dL	8–20 mg/dL
Creatinine	1.14 mg/dL	0.88 mg/dL	0.65–1.07 mg/dL
C-reactive protein	0.15 mg/dL	-	0–0.14 mg/dL

**Figure 2:**
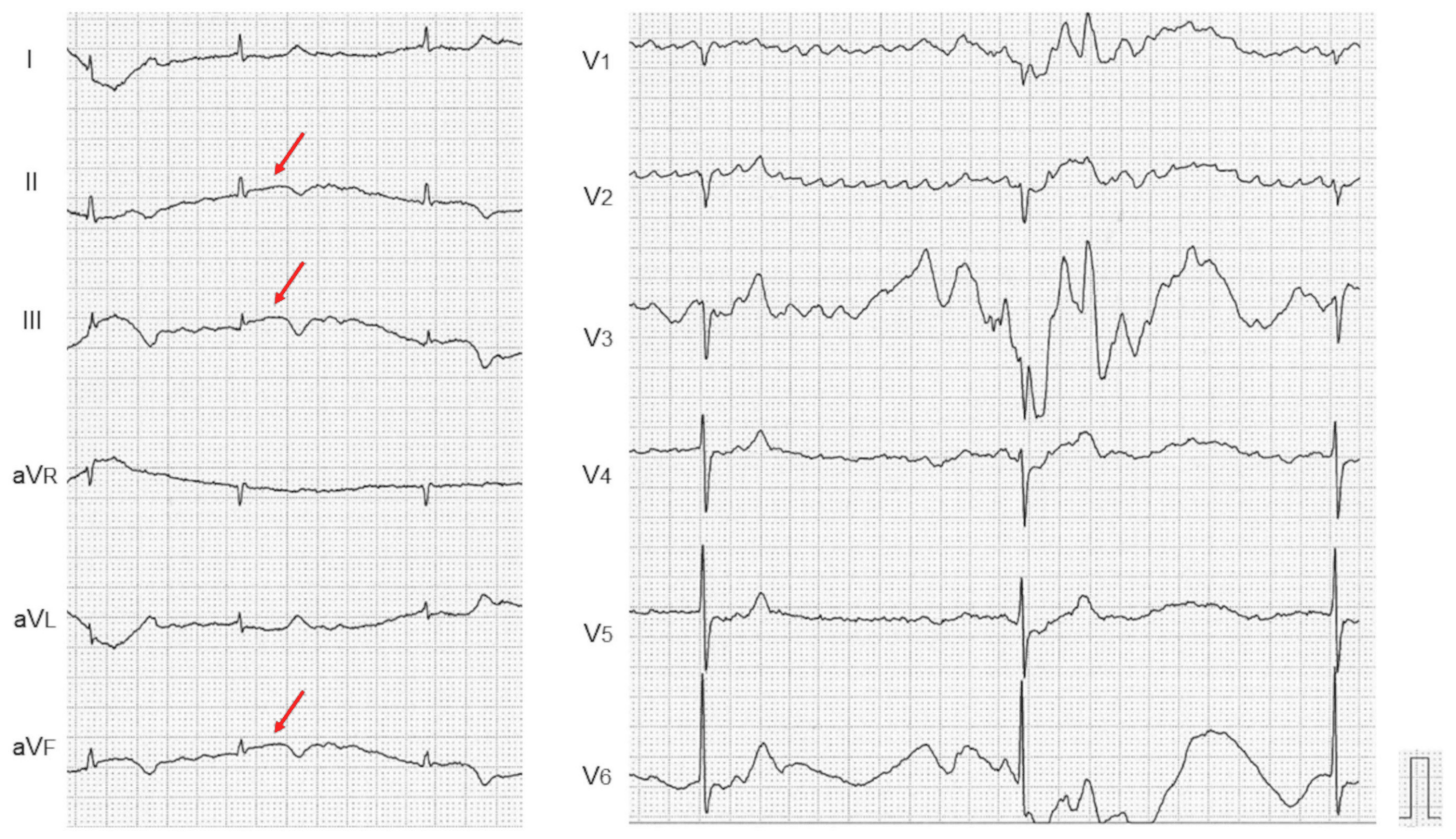
Electrocardiogram. An electrocardiogram showed atrial fibrillation with ST-segment elevations in II, III, and aV_F_ leads, although its quality was low due to cognitive problems (arrows). Complete atrioventricular block identified by regular RR interval was seen intermittently.

An electrocardiogram (ECG) showed AF with ST-segment elevations in II, III, and aV_F_ leads although its quality was low due to cognitive problems (Figure 2, arrows). Complete atrioventricular block (CAVB) identified by regular RR interval was seen intermittently.

**Figure 3:**
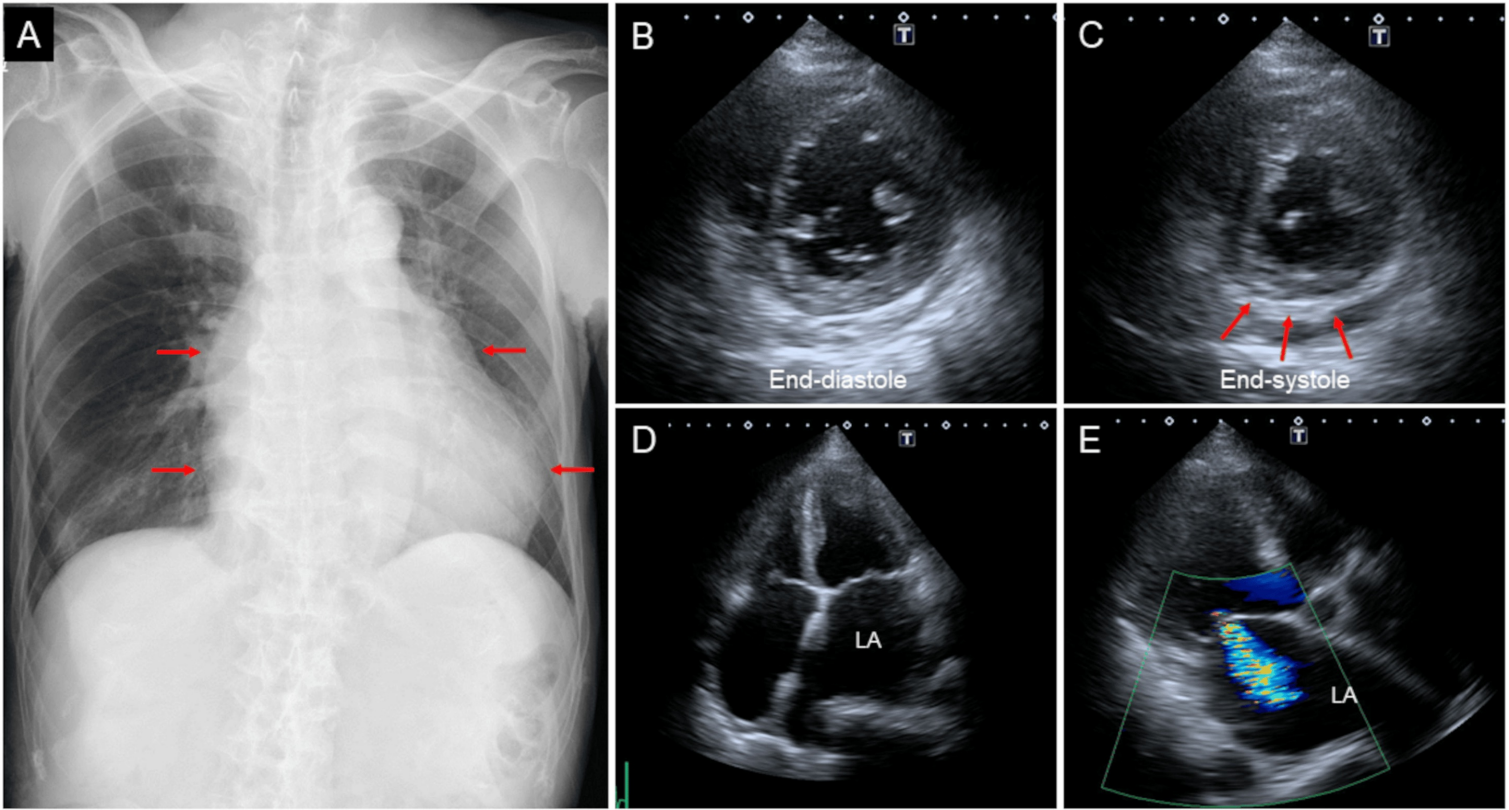
Chest radiograph and transthoracic echocardiographic images. A chest radiograph showed an enlarged cardiac silhouette with a cardiothoracic ratio of 62% (A, arrows). A transthoracic echocardiography revealed severe hypokinesia in the inferior area corresponding to ECG findings (B-C, arrows). LA was enlarged with moderate mitral regurgitation (D-E). LA: left atrium

A chest radiograph showed an enlarged cardiac silhouette with a cardiothoracic ratio of 62% (Figure 3A, arrows). A transthoracic echocardiography revealed severe hypokinesia in the inferior area corresponding to ECG findings (Figures 3B-3C, arrows). The left atrium was enlarged with moderate mitral regurgitation (Figures 3D-3E).

**Figure 4:**
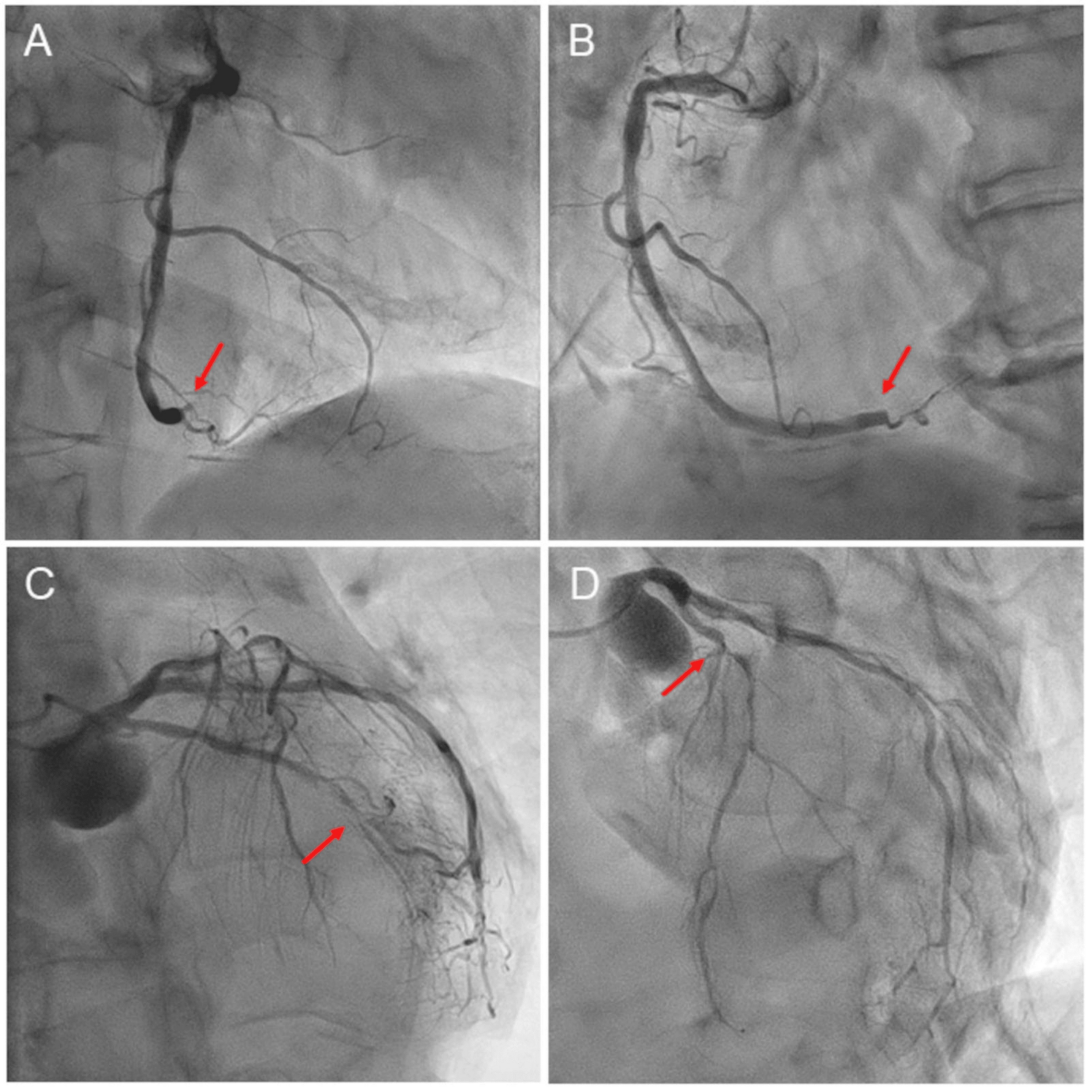
Right and left coronary angiograms. Emergency coronary angiography revealed a total occlusion of the distal right coronary artery (A-B, arrows). There were severe coronary artery diseases in the proximal left anterior descending artery and the mid-left circumflex artery (C-D, arrows).

Emergency coronary angiography revealed a total occlusion of the distal right coronary artery (Figure 4A-4B, arrows). There were severe coronary artery diseases in the proximal left anterior descending artery and the mid-left circumflex artery (Figure 4C-4D, arrows). A diagnosis of inferior AMI was made. Given that the thrombus was located at the distal end, coronary intervention was not indicated.

**Figure 5:**
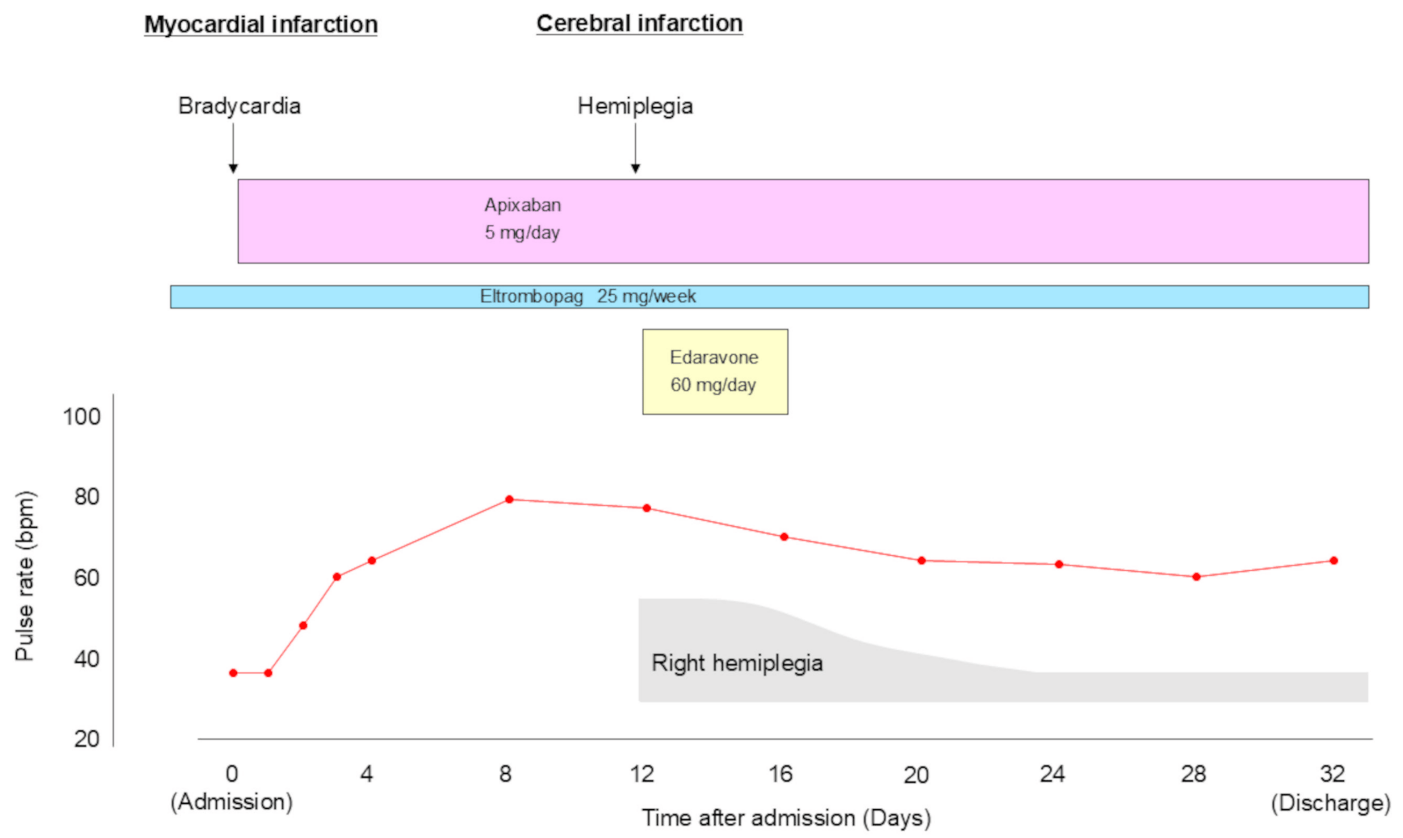
Clinical course after the onset of acute myocardial infarction. Apixaban (5 mg/day), a direct oral anticoagulant, was initiated, whereas antiplatelet therapy was not introduced. Severe bradycardia due to complete atrioventricular block was resolved within two days, suggesting reperfusion of the occluded right coronary artery. The patient’s heart rate gradually returned to around 60 beats/minute four days after the initiation of apixaban.

Apixaban (5 mg/day), a direct oral anticoagulant, was initiated, whereas antiplatelet therapy was not introduced. Severe bradycardia due to CAVB was resolved within two days, suggesting reperfusion of the occluded right coronary artery (Figure 5). The patient’s heart rate gradually returned to around 60 beats/minute four days after the initiation of apixaban.

**Figure 6:**
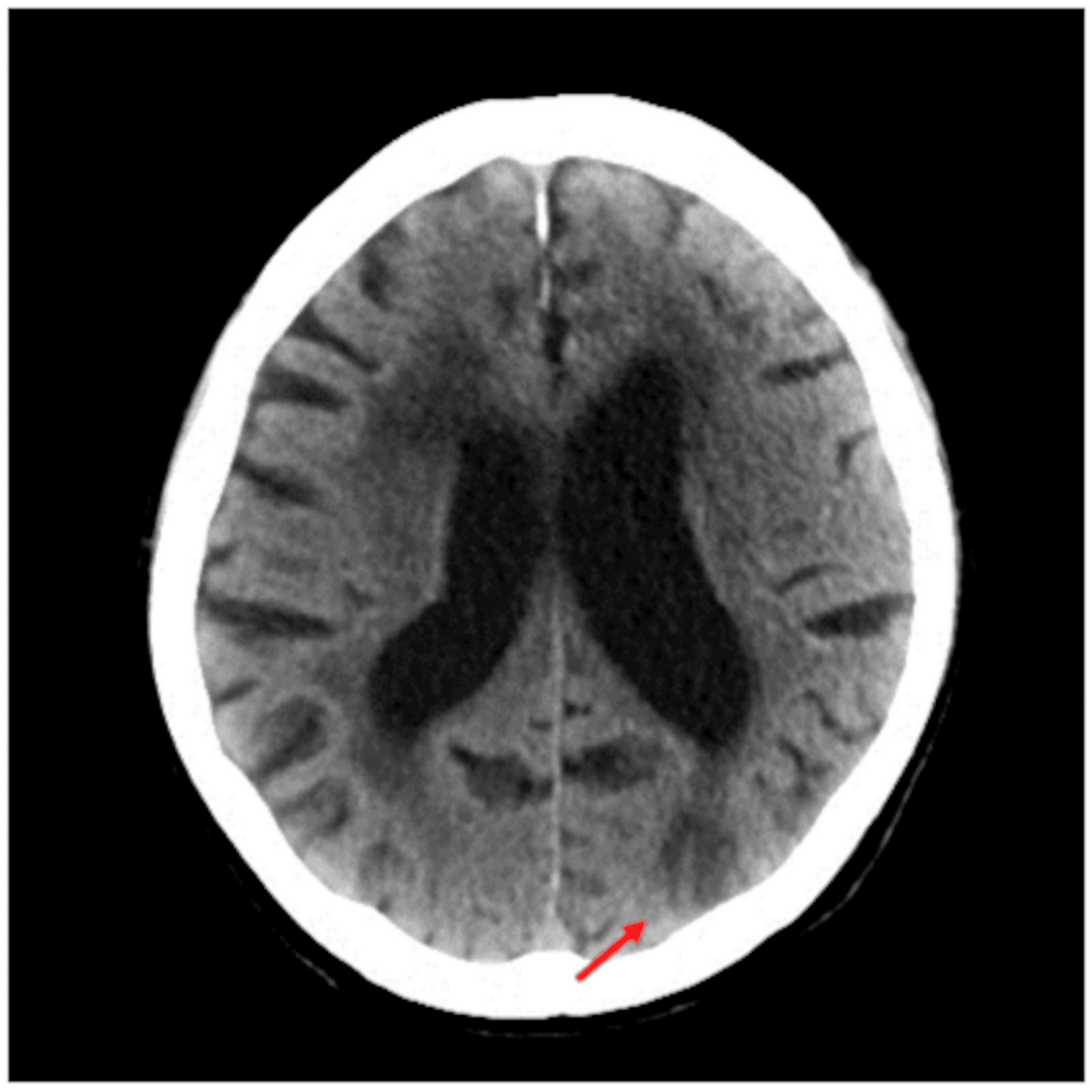
Computed tomography scan. A computed tomography scan revealed recurrent cerebral infarction with newly developed multiple low-density areas in the left parietal and occipital lobes (arrow).

Twelve days after admission, hemiplegia in the right upper extremity was found incidentally during rehabilitation, although the patient had no complaints. A computed tomography scan revealed recurrent cerebral infarction with newly developed multiple low-density areas in the left parietal and occipital lobes (Figure 6, arrow).

The patient was treated with intravenous edaravone (60 mg/day), an antioxidant agent with a neuroprotective effect, for five days [7]. The patient returned to the previous hospital for rehabilitation 32 days later. During the six-month follow-up, the patient followed an uneventful course without further thrombosis or bleeding complications.

## Discussion

In this report, we presented a case of recurrent thrombosis, presenting as AMI and cerebral infarction, in a very elderly patient with dementia, AF, and ITP on eltrombopag treatment. Multiple comorbidities with thrombosis and bleeding risks posed a therapeutic dilemma. Conditions unique to the elderly, such as cognitive impairment or frailty, led to a delayed diagnosis.

TPO-RAs have been widely used for the treatment of ITP as a second-line therapy. They can promote megakaryocyte maturation and increase platelet production [1-3]. On the other hand, the introduction of TPO-RAs raised the issue of thrombosis. The RAISE study, a large randomized controlled trial on the safety and efficacy of eltrombopag, showed that 2% of patients receiving eltrombopag had thromboembolic events compared with none in patients on placebo [2]. The EXTEND study, an analysis of the safety and efficacy of long-term treatment with eltrombopag, reported that the rate of thromboembolic events (6%) such as AMI, cerebral infarction, or pulmonary embolism did not increase with treatment duration past one year [3]. A recent multicenter study showed that the response and side effects of eltrombopag were variable throughout the follow-up period [6]. This indicates that patients need to be closely monitored, especially in terms of thrombosis and bleeding risks. In the present case, the patient had his first cerebral infarction two months after the initiation of eltrombopag although he was followed up every one to four weeks. Considering the time course of platelet count, a rapid and excessive increase in platelet count was associated with the occurrence of thrombosis.

Five cases of AMI suggestive of an association with eltrombopag have been reported [8-12]. Their ages ranged from 31 to 67 years, durations of eltrombopag treatment from 1 to 12 months, and platelet counts from 107 × 10^3^ to 350 × 10^3^/µL. There appears to be no significant relationship between AMI development and these parameters. In these cases, patients did not have AF and were treated with antiplatelet therapy and percutaneous coronary intervention. In contrast to previous cases [8-12], our patient was very elderly and had preexisting AF without anticoagulation. Given the presence of AF, an embolic risk factor, in addition to angiographic findings, coronary artery embolism was highly likely to contribute to the development of AMI [13]. The speculation was supported by the fact that our patient subsequently developed recurrent cerebral infarction with newly developed multiple low-density areas. This is why the patient was preferentially treated with anticoagulant therapy for AMI. Because the patient had severe coronary artery diseases, there remained a possibility that coronary plaque rupture contributed to the occurrence of AMI. However, no antiplatelet therapy was administered considering the patient’s condition such as age, frailty, or ITP. Consequently, anticoagulant therapy resolved CAVB, while failing to prevent recurrent cerebral infarction. The coexistence of thrombosis and bleeding risks was a sticky situation. Physicians need to provide appropriate management on a case-by-case basis in patients with multiple comorbidities with thrombosis and bleeding risks.

The other unique aspect was that the occurrences of AMI and recurrent cerebral infarction were recognized based on objective findings rather than on the patient’s complaints. Cognitive impairment and frailty led to delayed diagnosis. Previous studies have shown that patients with AMI in the absence of typical chest pain are older than those with chest pain [14]. This case highlights the difficulty of diagnosing diseases in the elderly.

## Conclusions

We encountered a case of recurrent thrombosis, presenting as AMI and cerebral infarction, in a very elderly patient with dementia, AF, and ITP on eltrombopag treatment. Multiple comorbidities with thrombosis and bleeding risks posed a therapeutic dilemma. This case gives an insight into how to manage a practical therapeutic problem in such a case. Physicians need to understand conditions unique to the elderly, such as cognitive impairment, frailty, or quality of life, that influence treatment goals. These efforts can help provide health benefits while minimizing treatment risks, especially in elderly patients with multiple comorbidities.
